# **Positive end-expiratory pressure and left ventricular function are key factors affecting right ventricular to pulmonary arterial coupling**

**DOI:** 10.1186/s13613-024-01284-4

**Published:** 2024-04-02

**Authors:** Cristina Santonocito, Siddharth Dugar, Filippo Sanfilippo

**Affiliations:** 1 Department of Anaesthesia and Intensive Care, A.O.U. Policlinico-San Marco, site “Policlinico G. Rodolico”, Via S. Sofia N 78, Catania, 95123 Italy; 2 Department of Critical Care Medicine, Respiratory Institute, Cleveland Clinic, Cleveland OH, USA; 3 Cleveland Clinic Lerner College of Medicine, Cleveland OH, USA; 4 Department of General Surgery and Medical-Surgical Specialties, Section of Anesthesia and Intensive Care, University of Catania, Catania, Italy

## Dear editor

we read with great interest the large retrospective study by Bowcock and Colleagues [1] on the assessment of right ventricular (RV) to pulmonary arterial (PA) coupling in the critically ill patients, enrolling over 1.000 patients. The authors used a simplified approach to evaluate RV-to-PA coupling dividing the tricuspid annular plane systolic excursion (TAPSE) by the tricuspid regurgitant velocity (TRV), dividing the population into tertiles of TAPSE/TRV ratio. The authors advised that such parameter has potentially significant prognostic relevance. Indeed, from their results, it seems likely that the use of TAPSE/TRV ratio could improve the risk stratification in the intensive care unit (ICU) setting, with lower values suggesting RV decoupling of RV-to-PA terminating into RV decompensation and hence being associated with poorer outcomes.

Notably, among other scientific efforts, the authors should be applauded for performing a multivariate analysis, accounting not only for the RV but also considering the left ventricular (LV) function. Moreover, they also explored the prognostic relevance in several subgroups of ICU patients and suggested that the prognostication value of TAPSE/TRV might be stronger in the respiratory and septic subgroups, which deserves further discussion to enlighten conditions where RV-to-PA coupling is crucial.

In particular, the RV-to-PA coupling is certainly affected by the use of positive end-expiratory pressure (PEEP), but the application of PEEP may have a two-fold (and opposite) action, producing either an increase or a decrease in the pulmonary vascular resistances (PVRs), according to the lung recruitment [2]. Indeed, the effects of PEEP on PVRs depend on one hand on the compression generated on the peri-alveolar vessels, which is proportional to the applied PEEP and result in increased PVRs; conversely, PEEP may produce the so-called rectilinearization of extra-alveolar vasculature, with greater values of PEEP typically reducing the PVRs. As roughly half of the investigated cohort was invasively ventilated, we think that an exploratory analysis accounting for the PEEP levels may provide further hints on the value of the TAPSE/TRV parameter for the non-invasive estimation of RV-to-PA coupling on identifying patients at higher risk.

As second key-point, Bowcock and Colleagues [1] assessed the impact of LV systolic function on TAPSE/TRV ratio according to the values of ejection fraction (LVEF), using a cut-off of 50% to identify systolic dysfunction with a dichotomic approach. However, for its effects on the outcomes of ICU patients, it is increasingly apparent that LVEF would be better treated as a continuous variable; indeed, both severely impaired LV systolic function (LVEF < 25%) as well as high values of LVEF (> 70%, hyperdynamic LV function) are associated with poorer prognosis [3, 4]. We believe that further analysis may also consider describing LV systolic function with a continuous rather than dichotomic approach. In addition, as the authors elegantly showed, a higher proportion of patients in the low TAPSE/TRV tertile group had a likelihood of increased LV filling pressures (as estimated by the E/e’ ratio), which in turn is a relevant parameter for the outcome of ICU patients [5].

In summary, TAPSE/TRV ratio represents a simple parameter that can be quickly calculated at bedspace from an apical 4-chamber view, without the need for values of PA systolic pressure. Hence, we think that Bowcock and Colleagues should be highly congratulated for their pioneer investigation, and their research “*lets the dance begin”* on the assessment of RV-to-PA coupling in the ICU setting; however, more insights should be considered by large prospective, and possibly multicentric, studies.

## Data Availability

Not applicable.
